# The time squares sequences: a new task for assessing visuospatial working memory

**DOI:** 10.3389/fnbeh.2023.1165906

**Published:** 2023-05-30

**Authors:** Pierandrea Mirino, Sara Mercuri, Anna Pecchinenda, Maddalena Boccia, Andrea Di Piero, Marta Soldani, Cecilia Guariglia

**Affiliations:** ^1^Department of Psychology, “Sapienza” University of Rome, Rome, Italy; ^2^Ph.D. Program in Behavioral Neuroscience, “Sapienza” University of Rome, Rome, Italy; ^3^Cognitive and Motor Rehabilitation and Neuroimaging Unit, IRCCS Santa Lucia, Rome, Italy

**Keywords:** working memory, temporal variation, visuospatial memory, cerebellum introductions, inferior olivary complex

## Abstract

**Introduction:**

Several studies have shown that the working memory is sensitive to temporal variations. We used a new visuospatial working memory task, the “Time Squares Sequences,” to investigate whether implicit variations in stimuli presentation time affect task performance.

**Methods:**

A total of 50 healthy participants saw two sequences (S1 and S2) of seven white squares presented in a matrix of gray squares and assessed whether S2 matched S1. There were four conditions depending on the spatial position and the presentation time (i.e., timing) of the white squares in S1 and S2: two with the same (S1 fixed/S2 fixed and S1 variable/S2 variable) and two with different (S1 fixed/S2 variable and S1 variable/S2 fixed) presentation times.

**Results:**

Findings showed impaired performance when S1 had a fixed presentation time and S2 had a variable presentation time.

**Conclusion:**

These findings are attributed to increased cognitive load due to S2 timing difference, pointing to a monitoring process, sensitive to temporal variations.

## Introduction

Every day we store and recall environmental information (e.g., writing down a phone number, making a mental shopping list, following the directions of a navigator along a route, and so on) and this storing and recalling activity relies on the working memory (WM): a multicomponent system that allows the integration and processing of a limited number of information for a short period of time (Baddeley and Hitch, 1974; Miller and Buschman, 2015). While working memory has limited capacities in terms of the amount of information that it can retain and its retention duration (Cowan, 2008, 2009; Cowan and Rouder, 2009), it allows for making calculations and solving tasks, and assists executive functions, including selective attention, reasoning, and planning (Cowan, 2014; Tang et al., 2019).

One of the components of the working memory is the visuospatial sketchpad (Baddeley and Hitch, 1974), described as the subsystem responsible for maintaining and processing visuospatial information. Accordingly, the visuospatial working memory consists of two separate subcomponents that encode, respectively, the perceptual characteristics of stimuli (shape and color) and their spatial position (Logie and Marchetti, 1991; Peterson, 2020). Different factors can influence how stimuli are encoded and stored in the visuospatial working memory; among these are salience, contextual information, stimulus quantity and position, binding, shape, and temporal duration (Emrich and Ferber, 2012; Pan and Luo, 2012; Souza and Oberauer, 2014; van Ede et al., 2017). In the present study, we will focus on the role attributed to the visuospatial working memory in processing temporal characteristics of the stimuli, which according to Mammarella et al. (Mammarella et al., 2013) requires an additional distinction for simultaneous and sequential presentation modality (Peterson, 2020). The first refers to spatial locations presented simultaneously in a working memory task and is related to static processes (Pickering et al., 2001), whereas the sequential modality refers to spatial locations sequentially presented so that individuals must recall each previous position. This is a dynamic process (Pickering et al., 2001). This distinction entails that the temporal characteristics of environmental stimuli are encoded and retained in the working memory to define *a temporal continuum of events*, to sequence the elements that are scattered along a path, to keep track of when a stimulus enters our perceptible space and then leaves it, to consider the temporal distance between one stimulus and another etc. Therefore, like other cognitive functions, working memory operates in a context in which time is an essential characteristic and this applies to visual, auditory, and tactile modalities (Wu et al., 2010; Souza and Oberauer, 2014). There are several accounts for the correlation between working memory and temporal characteristics of stimuli. For instance, for Bunting and Cowan’s model (Bunting and Cowan, 2005; Cowan, 2008), it is the passing of time that leads to a progressive deterioration of the working memory, while for other models, it is a distracting stimulus that affects participants’ performance during the tasks (Oberauer et al., 2012; Manohar and Husain, 2016). In contrast to these models that see working memory as being affected by the overall duration of the task (Harrison et al., 2015), models on temporal distinctiveness predict that working memory depends on relative time—that is, the time elapsed since the event to be recovered versus the time elapsed by other potentially interfering events (Souza and Oberauer, 2014). In fact, Oberauer and Lewandowsky showed that representations of different events overlap with each other to the degree that they are similar to each other and occur in close temporal proximity (Oberauer and Lewandowsky, 2013). Another aspect that affects working memory is the rate of stimuli presentation (i.e., the rhythm of a sequence). Some studies show that different rhythms of stimuli presentation compromise participants’ performance due to the unpredictability of events (Lecerf and De Ribaupierre, 2005; Lupo et al., 2018). For instance, Lupo et al. (2018) conducted a study with 71 healthy subjects who completed sequential and simultaneous navigation tests using an innovative sensor platform. They found that the variation in visual stimuli presentation times, without changes in interstimulus intervals, affects performance compared to when presentation times remain unchanged. However, although stimulus temporal unpredictability and stimulus duration both lead to false expectations, they are associated with different brain activations (Liu et al., 2008; Wu et al., 2010; Manohar and Husain, 2016; van Ede et al., 2017). In a recent review of fMRI studies, Mirino et al. (2022) concluded that the principal olivary nucleus (Lewis and Miall, 2003; Liu et al., 2008; Wu et al., 2010) plays a role in detecting temporal irregularity and proposed that this brain area could have a critical role in processing the temporal characteristics of visuospatial information.

In this context, it becomes important to understand the mechanisms underlying the effects of temporal regularity on visuospatial working memory. Therefore, the present study aimed at investigating whether temporal anomalies in the presentation of visual stimuli affect performance at a visuospatial working memory task, when the temporal variation is not part of the response, and it is not part of the attentional set.

We created a computerized Time Squares Sequence, which consists of two sequences of seven white squares, presented in a matrix of gray squares (S1 and S2). Participants responded based on whether the positions of all squares in the second sequence were the same as those in the first sequence. Importantly, we varied both the positions (same and different) and the presentation time of the squares (with and without temporal anomaly) of the two sequences (S1 and S2), leaving the interval between stimuli unchanged. We first report the findings from a pilot study, based on which the number of squares to use in the main study was established, followed by the findings of the main study.

## Materials and methods

### Participants

#### Pilot study

A total of 11 young volunteers (six men and five women), aged between 22 and 25 years (mean = 23.91; *SD* = 0.94) who attended school for a number of years (mean = 15.29 and *SD* = 1.60), took part in the pilot study. Participants were pre-screened and could take part in the study only if they did not have a history of neurological and/or psychiatric pathologies or head trauma, substance abuse, and/or use of psychotropic drugs.

#### Main study

*A priori* power analysis was performed with G*Power v. 3.1, which established that with power set at 95%, effect size f set at 0.25, and alpha value set at 0.05, a minimum sample of 36 subjects was required. A total of 50 young volunteers took part in the study, two participants were excluded because they did not respond to more than 25% of the trials, two for PC problems during the task, and one because of a low score (4) at the Corsi span. This resulted in 45 participants (24 men and 21 women), aged between 20 and 30 years (mean = 23.40; *SD* = 2.23) who had attended school for a number of years (mean = 15.07; *SD* = 1.88).

The tests were administered between October 2021 and March 2022. The experimental protocol was approved by the ethics committee of the Institutional Review Board.

### The time squares sequences (TSS)

The TSS test consisted of a 5 × 5 grid of gray squares delimited by white lines. In each trial, a sequence of seven white squares was presented (S1). Each of the seven white squares could appear sequentially for a variable duration (300–1,500 ms), and once a square disappeared, it was immediately followed by the next one. The total duration of each sequence (S1) was equal to 6,300 ms, followed by an interval of 1,000 ms, after which S2 was presented. In 50% of the trials (120 trials), S2 was the same as S1, while in the other 50% of the trials (120 trials), the position of one of the white squares differed by one adjacent position in any of the four possible directions in the grid (up, down, right, or left) with regard to the position in S1. Which square position varied and in which of the four possible directions varied randomly across the task with the only restriction being that the number of variations for the square and for each of the four possible positions was equal. The change of position in S2 could occur at any of the seven possible squares/positions, with the restriction of an approximately equal number of trials with the change at each of the squares/positions. Therefore, of the 240 total trials, half had a variation in the position of one of the squares in S2 (i.e., 120 trials). As there were seven squares/positions in each S2, there were between 16 and 18 trials for each possible variation (i.e., 120 trials/seven positions = 17.14). Each trial consisted of two sequences—S1 and S2—where participants decided if the probe S2 matched S1. Unbeknownst to the participants, the sequence of the seven squares in S1 and S2 could be regular—with each square presented for 900 ms (fixed sequence)—or it could have a temporal anomaly, and each square was presented for a different duration (variable sequence). As an example of variable sequence, the seven squares could be presented for, 300 ms, 1,200 ms, 1,500 ms, 1,200 ms, 600 ms, 300 ms, or 1,200 ms. Therefore, the presentation time of the seven squares in the two sequences (S1 and S2) could be fixed or variable, and the position of the seven squares in the two sequences (S1 and S2) could be the same or it could be different regarding one of the squares. This resulted in four different conditions of 60 trials, each based on spatial position and temporal duration of the seven white squares in S1 and in S2:

1. S1: Fixed Sequence—S2: Fixed Sequence (FF)
2. S1: Fixed Sequence—S2: Variable Sequence (FV)
3. S1: Variable Sequence—S2: Fixed Sequence (VF)
4. S1: Variable Sequence—S2: Variable Sequence (VV).

**Figure 1:**
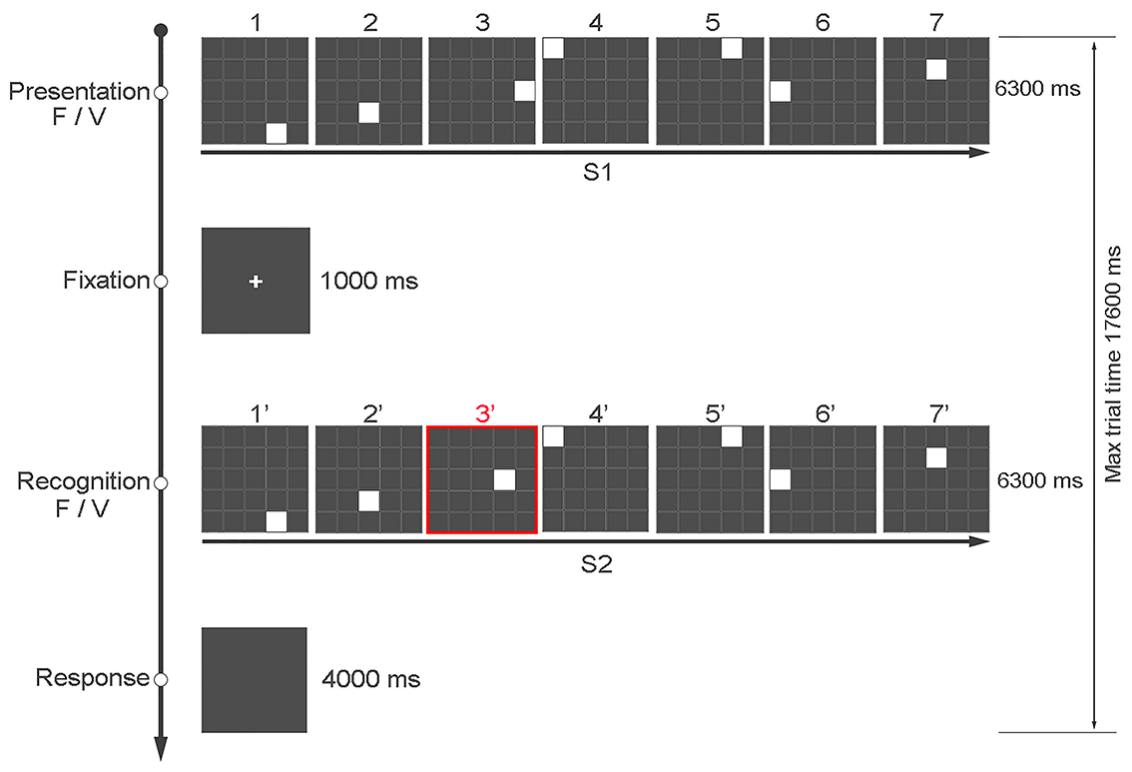
An example of the sequence of events in a typical trial of the “Time Squares Sequences” task, with the first and third rows showing the position and presentation time of S1 and S2, respectively. The figure shows an example in which the presentation time of the seven squares is fixed at 900 ms in both S1 and S2, but the position of square 3 in the matrix is different (from 300 ms to 1,500 ms, step 300 ms).

The task consisted of a total of 240 trials divided in two blocks of 120 trials each, presented in random order. Blocks were separated by a 3-min break. Each participant responded by pressing one of two keys of the keyboard based on whether the positions of the seven squares in S1 and S2 were the same (key “z”) or different (key “m”). The keys could only be pressed at the end of S2 up to a maximum of 4 s. To indicate to the participant when they could answer, the screen slightly changed shades of gray (see Figure 1).

We used Open Science Tools Ltd. (Peirce et al., 2019), which allows administering the task in the laboratory on a local PC, on the screen of the RMI machine, or remotely using an online platform via pavlovia.org server made available by the same organization.

### Pilot study

The TSS was presented on a 23” PC (Dell 2311Hf) monitor. The goal of the pilot study was to identify the number of squares to be used in the task that resulted in a performance accuracy of approximately 70–75% and that was neither too difficult nor too easy for our participants, thus avoiding ceiling or floor effects. Therefore, three versions of the TSS were used with five, six, or seven white squares, respectively, only for the condition FF (e.g., 40 trials, of which 20 were the same and 20 were different) and 10 trials, which were excluded from the analyses, with a different number of squares to avoid the habituation effect. Each participant completed the three versions of the task, which were presented in a new random order to each participant.

### Procedure

After providing informed consent, participants completed the pre-screening interview (see exclusion criteria in the Participants section) and the Corsi test (Spinnler and Tognoni, 1987). Only participants with a visuospatial memory span of 7 ± 2 (Miller, 1994) were invited to complete the task; for convergent validity, the Circle Task (Castillo et al., 2021) was also used to assess visuospatial working memory capabilities. Finally, the Repeatable Battery for the Assessment of Neuropsychological Status battery (RBANS) (Randolph et al., 1998) was used as a general assessment of cognitive abilities. Upon completion of the pre-screening, participants completed the Time Squares Sequences. The presentation order of the four different conditions was randomized between subjects. The overall duration of the experiment was 90 min.

### Data analyses

#### Pilot study

Accuracy data from the pilot study were analyzed with a one-way ANOVA with several squares (Span: 5, 6, 7) as a factor.

#### Main study

Five response types were computed:

1. HIT corresponds to “TRUE” responses when S1 and S2 were the same (the participant pressed the “z” key);
2. CR corresponds to “FALSE” responses when S1 and S2 differed (the participant pressed the “m” key);
3. FA corresponds to “TRUE” responses when the S1 and S2 were different (the participant pressed the “z” key);
4. MISS corresponds to “FALSE” responses when S1 and S2 were the same (the participant pressed the “m” key);
5. NO-RESP corresponds to no response (the participant did not press any of the keys) within the set time.

Based on these five response types, we computed: (a) an accuracy Index (AI) as (HIT +CR)/(total trial number/4), where 4 is the number of conditions (FF, FV, VF, and VV), which can vary from 0 to 1, (b) an overall Accuracy Index for every single subject, (c) the mean response time (RTs) computed for each response type (Hit, CR, Fa, and Miss) in each condition, and (d) an overall response time for a single subject.

Data from the main study were first analyzed with Block as a factor with SPSS Statistics 27 using a 2 × 2 × 4 mixed-factorial ANOVA with Sex (2: male, female) as the between-subject factor, Block condition (2: Block1, Block2), and Temporal Condition (4: FF, FV, VF, VV). If Block was not statistically significant as a main effect or in interaction with other factors, data were averaged across blocks and analyzed with a 2 × 4 mixed-factorial ANOVA with Sex (2: male, female) as the between-subject factor and Temporal Condition (4: FF, FV, VF, VV) as the repeated measure factor (see Supplementary material for additional analyses on data distribution). For convergent validity, multivariate regression was used to assess whether accuracy and response time at the Time Squares Sequences predicted the K-INDEX, which identifies the number of items present in working memory using false alarms and hit rates, and RT at Circle Task (Castillo et al., 2021).

## Results

### Pilot study

There was a significant effect of Span [*p* < 0.001; *F*_(2, 29)_ = 12.161]. Bonferroni-corrected comparisons showed performance was lower with Span 7 (*M* = 65.26%; *SD* = 10.45) compared to Span 5 (*M* = 84.09%; *SD* = 7.85), *p* < 0.001 and Span 6 (*M* = 80.25%; *SD* = 9.89), *p* = 0.003. Performance did not differ between Span 6 and Span 5, *p* > 0.99. Therefore, with five or six squares, the TSS was too easy as performance accuracy was 80% and above, while with seven squares, the task was more difficult, with a performance accuracy of 65%, which makes this version more suitable for our study.

### Main study

Results for accuracy measures (HIT, CR, FA, and MISS) with and without Block as a factor did not show any statistically significant main effects or interactions.

**Figure 2:**
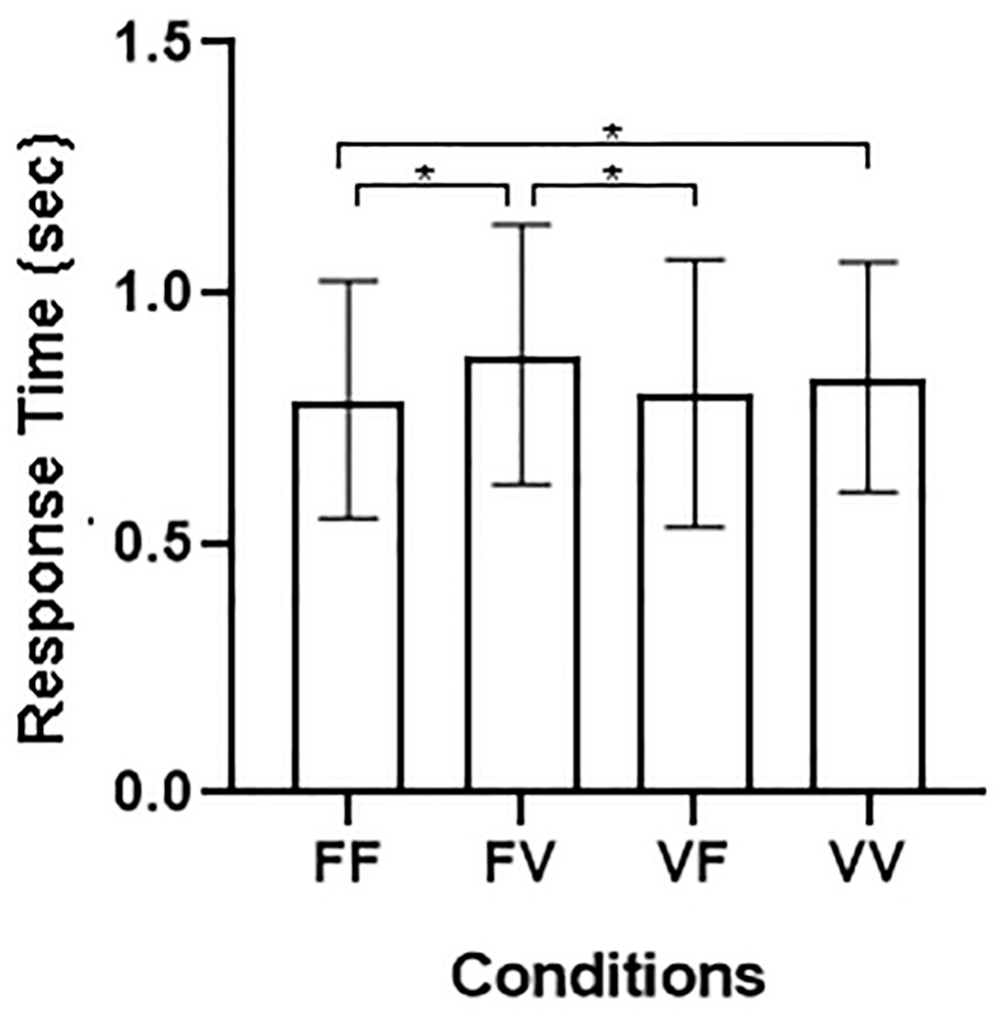
**(A)** Average RTs and SE for the four different conditions: FF, fixed sequence presentation—fixed sequence recognition; FV, fixed sequence presentation—variable sequence recognition; VF, variable sequence presentation—fixed sequence recognition; VV, variable sequence presentation—variable sequence recognition. **(B)** Individual performance distribution in the four conditions.

Results for RTs with Block as a factor showed a main effect of Temporal Condition [*p* < 0.001; *F*_(3, 129)_ = 10.21; *η*^2^ = 0.192], the main effect of Block [*F*_(3, 129)_ = 0.053, *p* = 0.818], the 2-way interactions Temporal Condition by Gender, [*F*_(3, 129)_ = 1.66, *p* = 0.179], Temporal Condition by Block, [*F*_(3, 129)_ = 1.00, *p* = 0.395], Gender by Block [*F*_(3, 129)_ = 0.08, *p* = 0.773], as well as the 3-way interaction Temporal Condition by Gender by Block [*F*_(3, 129)_ = 0.42, *p* = 0.738] were not statistically significant (Figure 2).

Results for RTs averaged across blocks showed a main effect of Temporal Condition [*F*_(3, 129)_ = 10.21, *p* < 0.001, *η*^2^ = 0.192], whereas the two-way interaction Temporal Condition by Gender, [*F*_(3, 129)_ = 1.66, *p* = 0.179], was not significant. Bonferroni-corrected pairwise comparisons showed slower responses on FV (*M* = 0.88; *SE* = 0.04), *p* < 0.001 and on VV trials (*M* = 0.83; *SE* = 0.04), *p* = 0.02 compared to FF trials (*M* = 0.78; *SE* = 0.04). In addition, responses on FV trails were also slower than on VF trials (*M* = 0.80; *SE* = 0.04), *p* < 0.003.

Regression analysis with AI (general accuracy index of Time Squares Sequences) as an independent variable and K-INDEX of Circle Task as a dependent variable show that AI predicts performance on the accuracy index Circle Task (*β* = 0.312; *t* = 2.228, *p* = 0.031). A second regression performed between overall RTs as an independent variable and RT-CIRCLE as a dependent variable showed that response times at TSS predict response times at Circle Task (*β* = 0.485, *t* = 3.361, *p* < 0.001) (Tables 1, 2).

**Table 1:** Accuracy index for the TSS task: the table shows minimum (min), maximum (max) values, mean and standard deviation (SD) of the Accuracy Index (AI), which can range from 0 to 1 for each condition.

	Min	Max	Mean	*SD*
AI_FF	0.47	0.87	0.65	0.089
AI_FV	0.52	0.90	0.65	0.093
AI_VF	0.45	0.88	0.65	0.093
AI_VV	0.52	0.92	0.66	0.092
AIm	0.56	0.87	0.65	0.078

AI_FF, Accuracy Index for FF Condition; AI_FV, Accuracy Index for FV Condition; AI_VF, Accuracy Index for VF Condition; AI_VV, Accuracy Index for VV Condition; AIm, general average Accuracy Index.

**Table 2:** RTs minimum (min), maximum (max) values, mean, and standard deviation (SD) for each condition of the TSS task.

	Min	Max	Mean	*SD*
RT_FF	0.43	1.35	0.79	0.24
RT_FV	0.49	1.49	0.88	0.26
RT_VF	0.40	1.59	0.80	0.27
RT_VV	0.44	1.42	0.83	0.23
RTm	0.45	1.45	0.85	0.24

RT_FF, average response time for FF condition; RT_FV, average response time for FV condition; RT_VF, average response time for VF condition; RT_VV, average response time for VV condition; RTm, general average response time.

## Discussion

The current study investigated to what extent temporal anomalies in stimuli presentation affect performance when the temporal variation is not part of the response or part of the attentional set.

We used a novel task and examined whether temporal anomalies of visuospatial stimuli with random and unpredictable spans could affect participants’ working memory abilities and, therefore, their performance. In doing so, we also provide a new tool to be used for working memory assessments.

The computerized “Time Squares Sequences” test allowed for the creation of a series of sequences of visuospatial stimuli, in which unbeknownst to participants, the presentation duration of the elements that made up the sequences could be the same (no anomaly) or varied (with anomaly). Participants responded based only on the spatial characteristics of the stimuli.

Our findings showed that response speed but not accuracy was affected by this manipulation, namely, responses were slower in the fixed–variable (FV) than in the fixed–fixed (FF) and the variable–variable (VV) conditions. Importantly, that response speed did not differ between the fixed–fixed and the variable–fixed conditions suggests that it is not the presence of an anomaly but rather the timing of the anomaly that affects performance. In fact, participants were more sensitive to the temporal anomaly when it was present during the recognition phase (i.e., FV and VV conditions), affecting stimulus processing and slowing down responses. This account is in line with a study by Blalock and Clegg (2010), in which the variation in participants’ performance was investigated using a task with simultaneous or delayed sequences of stimuli. Findings showed better performance accuracy in the condition with simultaneous presentation of stimuli. This is because when we store a sequence of events, adding a time variable can affect the encoding. In fact, the literature shows that the simultaneous processing of visuospatial stimuli has a lower attentional and mnemonic load, as stimuli can be viewed as a single pattern (Lupo et al., 2018). On the other hand, different presentation times interfere with the processes of memorization and recognition (Bharti et al., 2020). This is because, compared to a simultaneous presentation, a variable sequence yields a greater cognitive load because, in addition to processing the single stimulus, the time elapsing between stimuli of the entire sequence also needs to be encoded. This greater cognitive load is reflected in poorer performance.

In our study, the timing was always a variable being processed in the coding phase, both when the temporal stimuli presentation was the same, as well as when it was different. Our findings showed that a sudden change in the stimuli presentation timing (i.e., an anomaly) affected only response times when the anomaly occurred during the recognition process (i.e., when S2 was presented). This suggests a mechanism for detecting the changes between the previous and the information that had just been seen (e.g., memorization phase and recognition phase), which operates after the coding phase. This mechanism would allow us to monitor the progress of events over time. However, it is also possible that this mechanism works independently and that, by detecting temporal anomalies, it considers the information only if the stimulus has changed from the one previously encoded.

Importantly, the current findings could guide future research into assessing the anatomical-functional aspects underlying the ability to detect temporal variations of stimuli. Based on extant evidence from fMRI studies, we have proposed that the inferior olive complex has a critical role in processing the temporal characteristics of visuospatial information (Mirino et al., 2022). For instance, such a proposal matches evidence by Xu et al. (2006), who showed the involvement of subcortical areas, including the inferior olive complex, an area located in the brainstem, in processing temporal characteristics of stimuli. Accordingly, future studies could help understand whether the activation of the inferior olive complex, when processing or detecting temporal anomalies, modulates the activation of other brain structures.

In conclusion, the present findings show that the “Time Squares Sequences” may represent an effective tool to evaluate time processing in visuospatial working memory tasks and that it could be also used to investigate underlying neural mechanisms. Moreover, the task can be successfully used to assess changes occurring with aging, as it can provide useful information on how time is processed during the physiological aging of the individual’s brain as well as during pathological aging (e.g., neurodegenerative illnesses) or following brain injury from head trauma (Mirino et al., 2022).

## Data availability statement

The raw data supporting the conclusions of this article will be made available by the authors, without undue reservation.

## Ethics statement

The studies involving human participants were reviewed and approved by the Institutional Review Board of Sapienza University of Rome Department of Psychology (protocol number 0000272 of 17.02.2022).

## Author contributions

CG and PM: conceptualization, methodology, test implementation. PM, SM, ADP, and MS: data collection. PM and AP: data analysis. PM and SM: writing—original draft preparation. CG, AP, and MB: writing—review and editing. CG and AP: supervision. CG: project administration. All authors contributed to the article and approved the submitted version.

## Conflict of interest

The authors declare that the research was conducted in the absence of any commercial or financial relationships that could be construed as a potential conflict of interest.

## Publisher’s note

All claims expressed in this article are solely those of the authors and do not necessarily represent those of their affiliated organizations, or those of the publisher, the editors and the reviewers. Any product that may be evaluated in this article, or claim that may be made by its manufacturer, is not guaranteed or endorsed by the publisher.

## Supplementary material

The Supplementary material for this article can be found online at: https://www.frontiersin.org/articles/10.3389/fnbeh.2023.1165906/full#supplementary-material
