# Supplementary material for: The time squares sequences: a new task for assessing visuospatial working memory
Source: Front Behav Neurosci. 2023 May 30;17:1165906. doi: 10.3389/fnbeh.2023.1165906 (PMC10267982; doi:10.3389/fnbeh.2023.1165906)
Supplement: Supplementary file 1 [file Data_Sheet_1.docx]

**Supplementary Material**

We used the Kolomogorov-Smirnov test to check whether the data is normally distributed and the results indicate that normality is respected for FF (p=0.2), for FV(p=0.077) but not for VF(p < 0.001), VV (p=0.038).

We therefore performed non-parametric statistics by applying the Friedman's test. The same effects reported in the manuscript emerged. More specifically, results showed the following statistically significant effects (FT=26.387; p < 0.001) for Pairwise Comparisons FF-VF (FT= -0.178; p=0.514), FF-VV (FT=- 0.889; p=0.001), FF-FV (FT=-1.200; p<0.01), VF-VV (FT=-0.711; p=0.009), VF-FV (FT=1.022; p<0.001), VV- FV(FT=0.311; p=0.253).

According to Ghasemi et. al, (2012) based the Central-Limit Theorem, with large enough sample sizes (> 30 or 40), the violation of the normality assumption should not cause major problems. As our study has 50 participants we opted for reporting parametric statistics (i.e., R-ANOVA) in the main text.


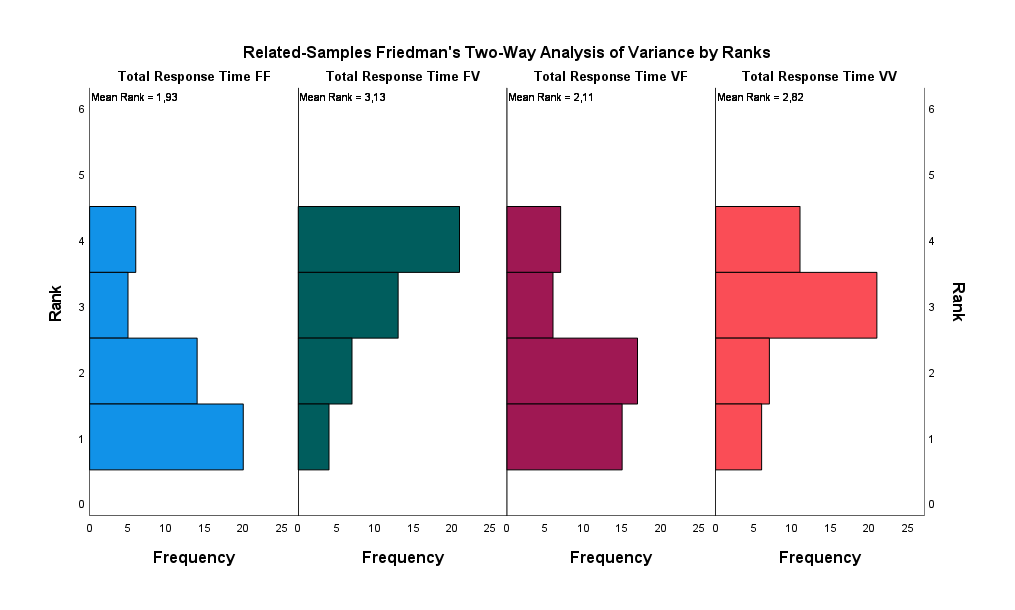


References

Reference; *Ghasemi A, Zahediasl S. Normality tests for statistical analysis: a guide for non-statisticians. Int J Endocrinol Metab. 2012Spring;10(2):486-9. doi: 10.5812/ijem.3505. Epub 2012 Apr 20. PMID: 23843808; PMC ID: PMC3693611.*
